# Genistein inhibits the release of pro-inflammatory substances from macrophages by suppressing potassium loss- and ROS-mediated caspase-1/gasdermin D pathway activation and pyroptotic cell lysis

**DOI:** 10.22038/ijbms.2024.77887.16854

**Published:** 2024

**Authors:** Meimei Yang, Tianqi Zhang

**Affiliations:** 1 The Fourth Affiliated Hospital of Harbin Medical University, Harbin, Heilongjiang 150081, PR China; 2 Department of Neurology, The Fourth Clinical Hospital of Harbin Medical University, Yiyuan Street 37, Harbin 150081, Heilongjiang, PR China

**Keywords:** Gasdermin D, Genistein, HMGB1, Inhibition, Pyroptosis, Release

## Abstract

**Objective(s):**

The expression of pro-inflammatory substances is closely related to various diseases. Genistein (GEN), a soy isoflavone, has been proven to inhibit the production of pro-inflammatory substances in macrophages. This study aimed to determine whether GEN exerts its inhibitory effect on the expression of pro-inflammatory substances by suppressing the release of these substances via attenuating pyroptotic cell lysis.

**Materials and Methods:**

Mice were treated with lipopolysaccharide (LPS) and GEN. J774A.1 cells were treated with LPS, adenosine triphosphate (ATP), and GEN. The expression of pro-inflammatory cytokines and high mobility group box 1 (HMGB1) was measured by qRT-PCR and ELISA. The activation of caspase-1 (CASP1) and cleavage of gasdermin D (GSDMD) were determined by Western blot assay. Lactic dehydrogenase (LDH) assay and CCK8 assay were performed to determine the integrity of the cell membrane and cell viability. The concentration of intracellular potassium (K^+^) and the production of reactive oxygen species (ROS) were determined by the colorimetric method and flow cytometry, respectively.

**Results:**

GEN inhibited the production of IL-1β and HMGB1 in LPS-challenged mice and LPS+ATP-treated mouse macrophages by inhibiting GSDMD-mediated pyroptotic cell lysis. Mechanistically, GEN could prevent the loss of intracellular K^+^ and the production of ROS caused by LPS+ATP treatment, thereby inhibiting the activation of CASP1. The pathological significance of the release of HMGB1 could be partially attributed to its ability to induce cell apoptosis.

**Conclusion:**

GEN inhibits CASP1/GSDMD-mediated pyroptotic cell lysis and the following release of pro-inflammatory substances by suppressing K^+^ loss and ROS production of macrophages.

## Introduction

Genistein (GEN) is an isoflavone widely existing in leguminous plants, seeds, and fruits of other plants, and vegetables (1). GEN is most often consumed by humans via soybeans or soya products (2), given that soybean is a major source of GEN (1). The health benefits of GEN have long been recognized. 

GEN has a variety of biological activities (3), which makes it a potential auxiliary medicinal agent against different types of diseases. Anti-inflammatory activity is crucial for GEN’s medicinal functions, as inflammation plays a broad role in the pathogenesis of various diseases (4). A large body of studies showed that GEN can suppress the mRNA transcription of pro-inflammatory substances such as interleukin-1β (IL-1β), IL-6, IL-8, tumor necrosis factor-α (TNF-α), and high mobility group protein B1 (HMGB1) by inhibiting intracellular signaling pathways, such as the nuclear factor kappa-light chain enhancer of the activated B cell (NF-κB)-related pathway (5-7). However, given that GEN can target a variety of biomolecules and several signaling pathways (8-10), there might be other ways by which GEN exhibits anti-inflammation activity.

Different from pro-inflammatory cytokines like IL-6, IL-8, and TNF-α, the release of IL-1 family cytokines (IL-1α, IL-1β, IL-18, and IL-33) and HMGB1 is not mediated by signal peptides but by leakages in the cell membrane or by cell lysis (11, 12). Gasdermin (GSDM) proteins are executioners of pyroptosis (13). The disjunction of the amino-terminal domain (NT) and carboxyl-terminal domain (CT) of GSDM protein by protease-mediated (such as caspases, granzymes, and viral and bacterial proteases) proteolytic cleavage releases the pore-forming activity of GSDM-NT, leading to pyroptotic cell lysis (13). Pores formed by GSDM-NTs in the cell membrane and the following cell lysis have been proven to be important ways for the release of IL-1 family cytokines and HMGB1 (11, 14). Inhibition of pyroptosis can effectively attenuate inflammation (15), which leads to the question of whether GEN exhibits inflammation-inhibitory activity by affecting pyroptosis. 

The current study showed that GEN could inhibit the expression of IL-1β and HMGB1 by suppressing their release from the cells. Mechanistically, GEN inhibited caspase-1 (CASP1)-mediated gasdermin D (GSDMD) cleavage by attenuating the loss of intracellular potassium (K^+^) and the production of reactive oxygen species (ROS) in lipopolysaccharide (LPS)+adenosine triphosphate (ATP)-treated mouse macrophages, thereby mitigating the pyroptotic lysis of the cells. The inhibition of pyroptotic lysis of macrophages by GEN hindered the release of IL-1β and HMGB1, thereby alleviating downstream events such as HMGB1-induced cell death of myeloid and non-myeloid cells. This study provides essential data for understanding the mechanism of GEN in treating inflammation-related diseases.

## Materials and Methods

***Animal studies***

Female C57BL/6 mice (18–20 g in body weight) were obtained from Changchun Yise Laboratory Animal Technology Co. (China). All animal care and procedures were approved by the Medical Ethics Committee of the Fourth Affiliated Hospital of Harbin Medical University (2021-WZYSLLSC-23). The mice were first treated with GEN (15 mg/kg body weight in 100 μl of 10% DMSO+90% corn oil) by intraperitoneal injection. After 2 hr, the mice were intraperitoneally injected with LPS (10 mg/kg body weight in 100 μl of sterile PBS). Mice were sacrificed 24 hr after LPS administration. Sera of the mice were collected for ELISA. The tissues were homogenized in RIPA lysis buffer (for Western blot) or fixed in 10% paraformaldehyde (for histopathological analysis).

***Cells and treatments***

J774A.1 cells (mouse monocyte/macrophage cell line), L929 cells (mouse fibroblast cell line), and MLE-12 cells (mouse alveolar epithelial cell line) were purchased from American Type Culture Collection and cultured in RPMI1640 culture medium or DMEM culture medium supplemented with 10% fetal bovine serum at 37 °C. 

The cells were seeded into a six-well cell culture plate at a density of 1×10^6^ cells/well and allowed to grow at 37 °C overnight. The cells were treated with any one or different combinations of lipopolysaccharide (LPS; 1 μg/ml), adenosine triphosphate (ATP; 5 mM), GEN (10–50 μM), KCl (25 or 100 mM), NaCl (25 or 100 mM), N-acetylcysteine (NAC; 1.25–10 mM), glycine (GLY; 5 mM), necrosulfonamide (NSA; 20 μM), and recombinant HMGB1 (rHMGB1; MCE, USA; 1 or 10 μg/ml) as indicated in the results.

After treatment, a CCK8 assay was performed to determine the viability of the cells. The culture medium was harvested for lactate dehydrogenase (LDH) content measurements. The cells were observed under an optical microscope or under a laser confocal microscope after immunostaining or subjected to ROS measurement. The total RNA or total protein of the cells was prepared. The collected samples were subjected to Western blot analysis, qPCR analysis, or K^+^ concentration measurement.

***Histopathological analysis***

The fixed tissues were embedded in paraffin and cut into 5 μm sections. H&E staining was performed. The slides were observed under a microscope, and the representative pictures were captured.

***Enzyme-linked immunosorbent assay***

The concentrations of IL-1β, IL-6, TNF-α, and HMGB1 were determined by using a QuantiCyto® Mouse IL-1β ELISA kit (NeoBioscience, China), QuantiCyto® Mouse IL-6 ELISA kit (NeoBioscience, China), QuantiCyto® Mouse TNF-α ELISA kit (NeoBioscience, China), and mouse HMGB1 ELISA Kit (Mlbio, China) according to the manufacturer’s instructions.

***Western blot***

The protein concentration of the samples was determined by the BCA method. Proteins (30 μg) from each sample were separated by sodium dodecyl sulfate-polyacrylamide gel electrophoresis and transferred to nitrocellulose membranes. The membranes were incubated overnight at 4 °C with anti-GSDMD (Abcam, USA), anti-CASP1 (Biolegend, USA), anti-CASP3 (CST, USA), anti-β actin (Zsbio, China), or anti-GAPDH (Zsbio, China) primary antibodies (1:1000 dilution). The membranes were then incubated with horseradish peroxidase-conjugated secondary antibody (1:5000 dilution) for 1 hr at room temperature. A BeyoECL Star kit (Beyotime, China) was used for detection.

***qRT-PCR***

Total cellular RNA was extracted with Trizol reagent and then transcribed into cDNA by using a reverse transcriptase kit. qRT-PCR was performed using the SYBR green method. The specific primer sequences for mouse IL-1β (16), IL-6 (17), TNF-α (17), and β-actin (16) are listed in Table 1. Values were normalized to β-actin expression level. The measurements were performed in triplicate. All expression changes were normalized to untreated controls.

***CCK8 assay***

Cells were treated as indicated in the results. After treatment, the culture medium was replaced with fresh RPMI1640 containing CCK-8 reagent, and incubation was carried out for 2 hr. The optical density (OD) of the cultures was measured at 450 nm by using a spectrophotometer. Data are shown as the percentage of the OD_450nm_ values of the treated group to that of the untreated group. 

***LDH assay***

The culture medium of the cells with different treatments was harvested. LDH content in the culture medium was measured using the LDH cytotoxicity assay kit (Beyotime, China) according to the manufacturer’s instructions. The LDH release rate was calculated using the following formula: percentage of LDH release = 100 × (experimental sample − culture medium background) / (maximum LDH release − culture medium background).

***ROS measurement***

The J774A.1 cells were treated as indicated. The intracellular ROS level of the cells was determined using a ROS assay kit (Beyotime, China) according to the manufacturer’s instructions. Measurement was performed by a flow cytometer. 

***Confocal microscope observation***

J774A.1 cells were treated as indicated and then fixed with polyformaldehyde. The fixed cells were permeabilized with Triton X-100. The GSDMD molecules in the cells were probed with an anti-GSDMD antibody (Affinity, China) and FITC-labeled goat anti-rabbit IgG. The cell membrane and nucleus were stained by a Cell Plasma Membrane Staining Kit with DiI (Beyotime, China) and DAPI, respectively. The cells were observed with a confocal microscope to determine the subcellular localization of GSDMD.

***Determination of intracellular K*** ^+^ ***concentrations***

J774A.1 cells were treated as indicated. The concentrations of intracellular K^+^ were determined by using a cellular potassium concentration quantitative test kit (chemical colorimetric; Shanghai Haling Biological Technology, China) according to the manufacturer’s instructions.

***Statistical analysis***

One-way ANOVA test was used in this study. The results are expressed as mean±SD. **** *P*<0.0001, ** *P*<0.01, * *P*<0.05, and ns not significant.

## Results

***GEN ameliorated LPS-induced tissue injury, reduced the expression of IL-1*** ***β*** ***and HMGB1, and inhibited the cleavage of GSDMD***

**Figure 1:**
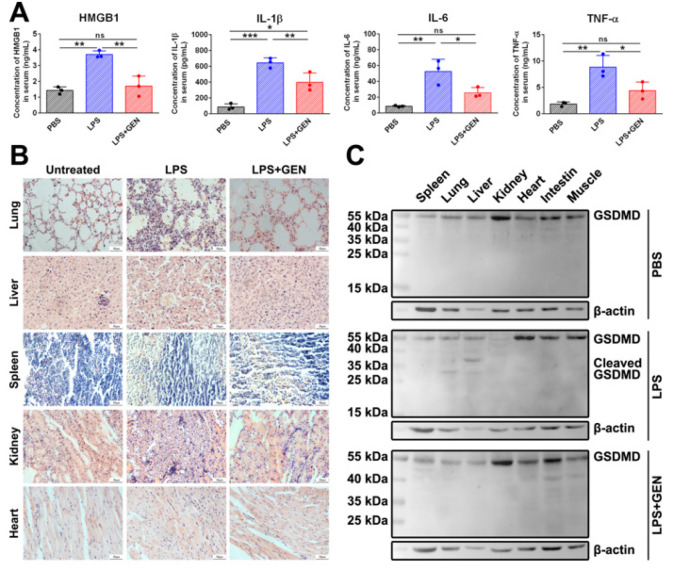
GEN alleviated LPS-induced overexpression of pro-inflammatory substances, histopathological changes, and GSDMD cleavage of mice

ELISA results showed that GEN treatment significantly inhibited the expression of IL-1β and HMGB1 in the tissues of mice (Figure 1A). Histological study showed that administration of GEN ameliorated LPS-induced tissue injury of the heart, lung, liver, and spleen (Figure 1B). Western blot results showed that GEN treatment inhibited the LPS-induced cleavage of GSDMD in tissues (Figure 1C). 

***Determination of the cytotoxicity of GEN***

**Figure 2:**
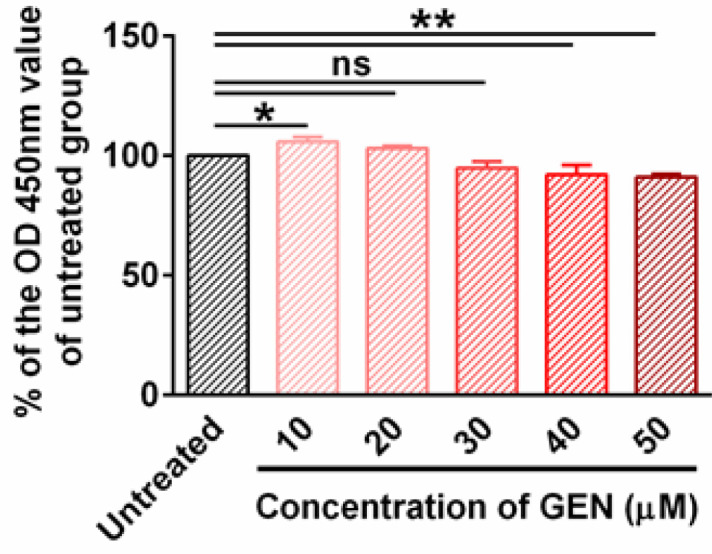
Determination of the dose-dependent cytotoxicity of GEN

Given that a monocyte/macrophage is a main source of pro-inflammatory cytokines, the following tests were performed on J774A.1 cells. We first measured the cytotoxicity of GEN in J774A.1 cells. CCK8 assay showed that 10, 20, and 30 μM GEN treatment did not cause a significant decrease in cell viability compared with PBS. By contrast, 40 and 50 μM GEN treatment led to a significant decrease in cell viability (Figure 2). Therefore, 30 μM was chosen as the working concentration of GEN in *in vitro* assays.

***Genistein inhibited LPS-induced expression of pro-inflammatory substances of macrophages at the transcription and post-translation level***

**Figure 3:**
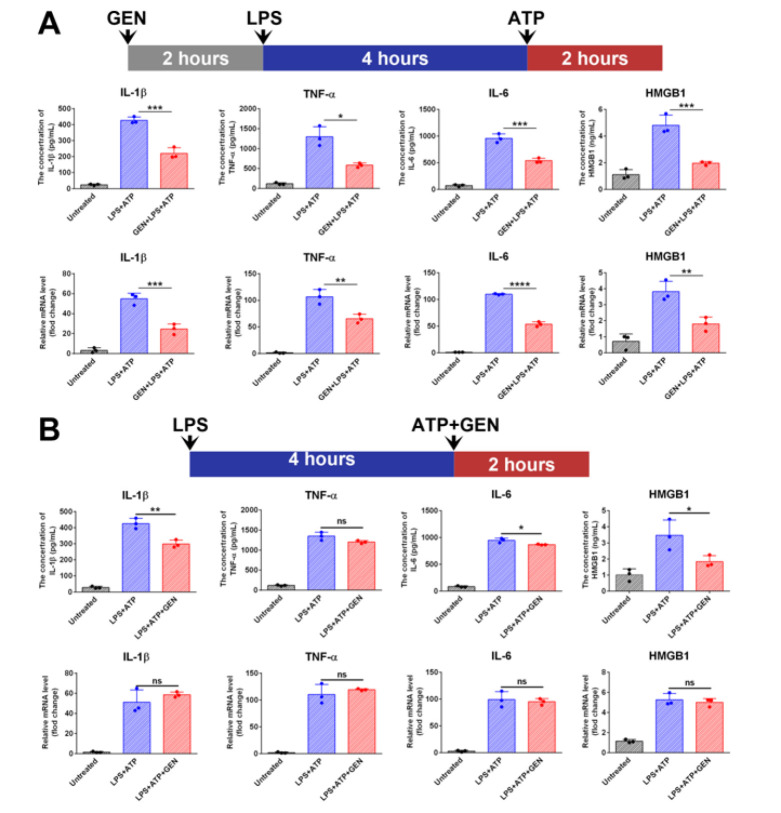
GEN inhibited the expression of IL-1β, IL-6, TNF-α, and HMGB1 at both the transcriptional and post-transcriptional levels

The expression levels of IL-1β, IL-6, TNF-α, and HMGB1 were significantly up-regulated in J774A.1 cells treated with LPS+ATP (Figure 3A). Pretreatment with GEN 2 hr before LPS priming significantly inhibited the expression of the pro-inflammatory cytokines and HMGB1 at the mRNA and protein levels (Figure 3A). When GEN was introduced after LPS priming and incubated with the cells together with ATP for 2 hr, the mRNA level of the cytokines and HMGB1 was not significantly affected (Figure 3B), nor was the protein level of TNF-α. However, the concentration of IL-1β and HMGB1 in the cultural medium of LPS+ATP+GEN-treated cells was significantly lower than that of the LPS+ATP-treated cells (*P*<0.01; Figure 3B). Although GEN co-treatment resulted in a significant decrease in the IL-6 concentration in the culture medium of LPS+ATP-treated cells (*P*<0.05), the decrease was not as significant as that of IL-1β and HMGB1 (Figure 3B). These results indicated that GEN could not only affect the transcription of mRNA of these pro-inflammatory substances but also affect the release of some of the pro-inflammatory substances.

The current work focused on determining the influence of GEN on the release of pro-inflammatory substances in the cells, so GEN was simultaneously added with ATP in the following experiments.

***GEN inhibited GSDMD-mediated pyroptosis of macrophages***

**Figure 4:**
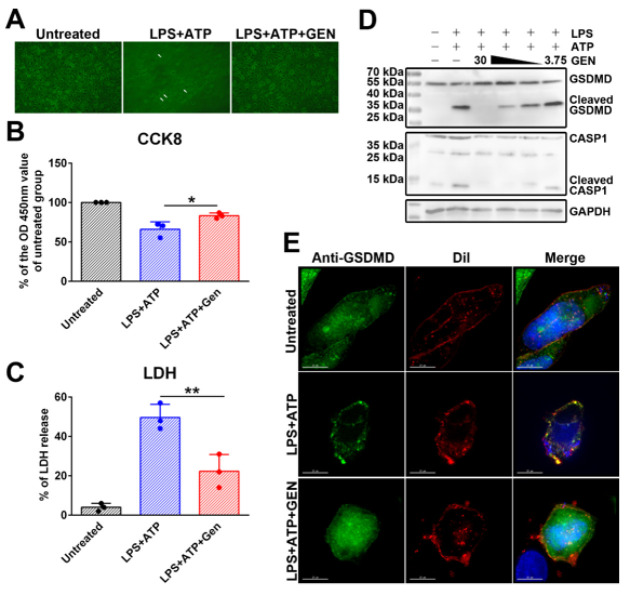
GEN attenuated CASP1/GSDMD-mediated pyroptosis

As shown in Figure 4A, LPS+ATP stimulation resulted in serious cell death of J774A.1 cells. The dead cells showed morphological characteristics of pyroptosis. GEN treatment significantly inhibited the cell death caused by LPS+ATP stimulation. CCK8 assay also showed that LPS+ATP stimulation led to a decrease in cell viability, and GEN treatment alleviated cell death (Figure 4B). Measurements of LDH in the culture medium indicated that LPS+ATP treatment promoted the release of LDH from the cells, and GEN treatment attenuated the LDH release caused by LPS+ATP treatment (Figure 4C). These results indicated that LPS+ATP treatment led to cell death by causing cell membrane damage, and GEN treatment might alleviate cell death by inhibiting cell membrane damage. 

Western blot assays showed that LPS+ATP stimulation led to CASP1 activation and GSDMD cleavage in J774A.1, and GEN inhibited these events in a dose-dependent manner (Figure 4D). Confocal microscopy observations after immunostaining of GSDMD-NT showed that LPS+ATP treatment led to the translocation of GSDMD to the cell membrane, and GEN treatment could inhibit this event (Figure 4E). Thus, GEN could inhibit the GSDMD-mediated pyroptosis of macrophages.

***GEN inhibited GSDMD cleavage by inhibiting potassium loss and ROS production of macrophages***

**Figure 5:**
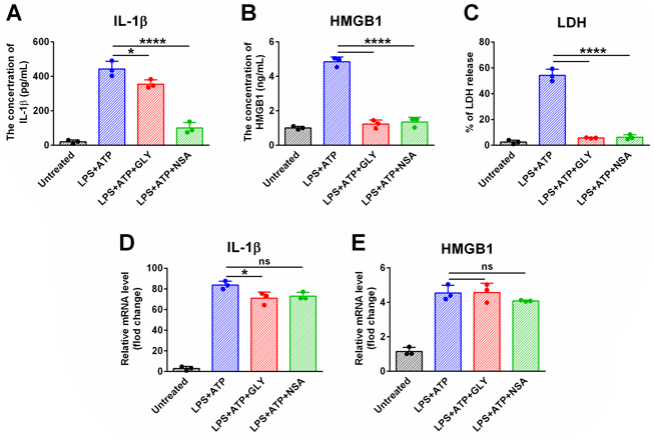
GEN inhibited LPS+ATP-induced CASP1 activation and GSDMD cleavage by inhibiting loss of intracellular K+ and the production of ROS

As shown in Figure 5A, LPS+ATP treatment caused a decrease in the intracellular potassium concentration, and GEN treatment attenuated LPS+ATP-induced potassium loss. When KCl (50 mM) was added into the culture medium, LPS+ATP-induced CASP1 activation and GSDMD cleavage were almost completely abolished, whereas NaCl (50 mM) only partially inhibited LPS+ATP-induced GSDMD cleavage (Figure 5B). These results indicated that the inhibition of GSDMD cleavage by GEN was related to the inhibition of potassium loss by GEN.

Flow cytometry analysis showed that LPS+ATP treatment increased ROS levels in J774A.1 cells and GEN treatment attenuated the increase in ROS level caused by LPS+ATP treatment (Figure 5C). To determine the significance of the inhibition of ROS production by GEN, we enrolled NAC (a scavenger of ROS) in the current work. The results showed that NAC could partially inhibit the activation of CASP1 and the cleavage of GSDMD caused by LPS+ATP treatment. These results indicated that the inhibition of GSDMD cleavage by GEN was related to the inhibition of ROS production by GEN (Figure 5D).

**Table 1:** Primers used to perform qRT-PCR

Target	Forward primer	Reverse primer
β-actin	GGAGGGGGTTGAGGTGTT	GTGTGCACTTTTATTGGTCTCAA
IL-1β	TGGCAACTGTTCCTG	GGAAGCAGCCCTTCATCTTT
TNF-α	ATAGCTCCCAGAAAAGCAAGC	CACCCCGAAGTTCAGTAGACA
IL-6	TGGAGTCACAGAAGGAGTGGCTAAG	TCTGACCACAGTGAGGAATGTCCAC

(A) Mice receiving LPS (10 mg/kg body weight) intraperitoneally have a significantly higher serum concentration of IL-1β, IL-6, TNF-α, and HMGB1 than the untreated mice. GEN pretreatment 2 hr before LPS injection significantly inhibited the expression of these proinflammatory substances induced by LPS stimulation. (B) LPS stimulation caused infiltration of inflammatory cells in the lung and kidney, loss of alveolar structure and alveolar wall thickening, and necrosis-like cell death of the liver, and GEN pretreatment alleviated the histopathological changes caused by LPS. (C) LPS stimulation caused the cleavage of GSDMD in the lung and liver. The decrease in the amount of full-length GSDMD in the kidney was observed, although the cleavage fragment of GSDMD was not observed on the blot. GEN pretreatment almost completely abolished the LPS-induced GSDMD cleavage. (**** *P*<0.0001, *** *P*<0.001,* *P*<0.05, ns, not significant)

J774A.1 cells were treated with different concentrations of GEN (10 to 50 μM) for 2 hr. The viability of the cells was determined by CCK8 assay. The results were presented as the percentage of OD450nm values of GEN-treated groups to that of the untreated group. 10 μM GEN caused a significant increase in cell viability (* *P*<0.05). 20 μM and 30 μM GEN did not significantly affect the viability of the cells (ns not significant). 40 μM and 50 μM GEN treatment reduced the viability of the cell significantly (** *P*<0.01). GEN: Genistein

(A) J774A.1 cells were seeded into a six-well cell cultural plate. GEN was added into the culture medium to a final concentration of 30 μM. Two hours later, LPS was added to a final concentration of 1 μg/ml. Four hours later, ATP was added to a final concentration of 5 mM. Two hours later, the cells and culture medium were harvested separately. mRNA level of IL-1β, IL-6, TNF-αand HMGB1 was determined by qRT-PCR and protein concentration of IL-1β, IL-6, TNF-α and HMGB1 in the culture medium was determined by ELISA. The results showed that LPS treatment up-regulated the expression of IL-1β, IL-6, TNF-α, and HMGB1 at both mRNA and protein levels. GEN pretreatment significantly inhibited LPS-induced expression of these proinflammatory substances at both mRNA and protein levels (**** *P*<0.0001, *** P<0.001, ** *P*<0.01, * *P*<0.05). (B) J774A.1 cells were treated with LPS (1 μg/ml) for 4 hr. Then, GEN (30 μM) and ATP (5 mM) were added to the culture medium. Two hours later, the cells and culture medium were harvested separately. qPCR results showed that LPS treatment up-regulated the transcription level of IL-1β, IL-6, TNF-α, and HMGB1 mRNA. GEN +ATP treatment did not affect the quantity of IL-1β, IL-6, IL-8, and HMGB1 mRNA significantly, but caused a significant reduction of the concentration of IL-1β, IL-6, and HMGB1 protein in the culture medium. The protein concentration of TNF-α was not significantly affected by GEN. (** P<0.01,* *P*<0.05, ns not significant).

(A) LPS+ATP stimulation induced death of J774A.1 cells. Some of the cells showed morphological changes like rounding, swelling, and loss of intracellular content (white arrows), which have been considered as the characteristics of pyroptosis. (B) CCK8 assay showed that LPS+ATP stimulation caused a significant decrease in cell viability, and GEN treatment mitigated the decrease in cell viability caused by LPS+ATP stimulation. (* *P*<0.05) (C) LDH assays showed that LPS+ATP stimulation induced LDH release of the cells, and GEN treatment partially inhibited LPS+ATP-caused LDH release. (** *P*<0.01). (D) LPS+ATP stimulation caused CASP1 activation and GSDMD cleavage in the cells. GEN inhibited CASP1 activation and GSDMD cleavage in a dose-dependent manner. 30 μM GEN almost completely abolished the two events, while 3.75 μM GEN showed no inhibitory effect. (E) J774A.1 cells were primed with 1 μg/ml LPS for 4 hr, then stimulated by ATP (5 mM) with or without GEN. The cells were fixed by 4% polyformaldehyde for 15 min at room temperature. Then the cells were treated with 0.1% Triton X-100 at room temperature for 30 min. Then the cells were immunostained with 1:200 diluted rabbit-anti-GSDMD antibody (which could recognize the NT domain of GSDMD according to the manufacturer) and FITC-labeled goat anti-rabbit IgG (Green). Then the cell membrane was stained by Dil,(Red) and the nucleus of the cells was stained by DAPI (Blue). Confocal microscope observation showed that GSDMD was located in the plasma of the cells. LPS+ATP stimulation resulted in the localization of GSDMD to the cell membrane, and GEN inhibited the change in the subcellular localization of GSDMD. GSDMD: gasdermin D; CASP1: caspase-1; LPS: lipopolysaccharide; ATP: adenosine triphosphate; LDH: lactate dehydrogenase

(A) J774A.1 cells were treated with LPS for 4 hr. Then, GEN (30 μM) and ATP (5 mM) were added into the culture medium and let to incubate with the cells for 2 hr. Concentration of intracellular K+ was measured. LPS+ATP stimulation caused significant decrease in concentration of intracellular K+, and GEN suppressed the reduction of intracellular K+ (**** indicates *P*<0.0001, * indicates *P*<0.05) (B) Adding KCl (100 mM or 25 mM) into the culture medium at the same time with ATP, completely abolished LPS+ATP-induced CASP1 activation and GSDMD cleavage. Extracellular NaCl (100 mM or 25 mM) only partially inhibited CASP1 activation and GSDMD cleavage in LPS+ATP-treated cells. (C) Flow cytometry results showed that LPS+ATG stimulated the production of ROS in J774.1 cells, and GEN inhibited the production of ROS induced by LPS+ATP treatment. (D) J774A.1 cells were stimulated with LPS for 3 hr, then NAC was added into the culture medium and incubated with the cells for 1 hr. Then ATP was added and let to incubate with the cells for another 2 hr. The results showed that NAC inhibited CASP1 activation and GSDMD cleavage in LPS+ATP-treated J774A.1 cells in a dose-dependent manner.

**Figure 6:**
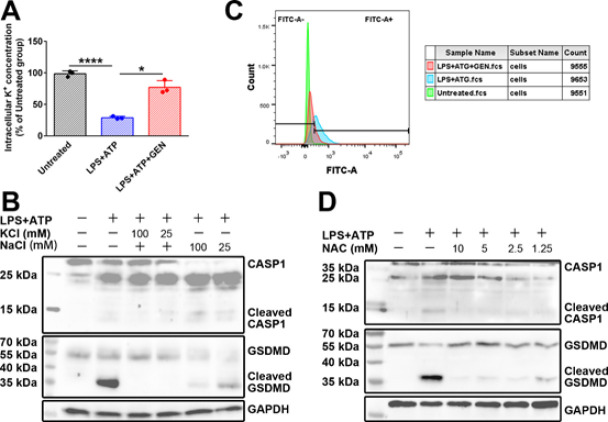
Inhibition of pyroptotic cell lysis suppressed the production of IL-1β and HMGB1

J774A.1 cells treated with LPS. Four hours later, GLY was added into the culture medium at the same time as ATP. NSA was added into the culture medium 3 hr later and let to incubate with cells for 1 hour, and then ATP was added into the culture medium. The cells were further incubated at 37 ºC for another 2 hr. The concentration of IL-1β (A) and HMGB1 (B) in the culture medium was determined by ELISA. The results indicated that the production of both IL-1β and HMGB1 was closely related to pyroptotic cell lysis since preventing cell lysis by GLY and NSA significantly reduced the amount of IL-1β and HMGB1 in the culture medium. GLY showed similar potential to NSA in inhibiting the production of HMGB1 but was less potent in inhibiting the production of IL-1β than NSA (**** *P*<0.0001, * *P*<0.05). Measurement of LDH in the culture medium showed that GLY and NSA significantly inhibited the release of LDH induced by LPS+ATP stimulation (C). The mRNA level of IL-1β and HMGB1 was determined by qPCR. The results showed that GLY caused reduction of the mRNA level of IL-1β (D), and did not affect the mRNA level of HMGB1 (E). NSA did not affect the mRNA level of either IL-1β (D) or HMGB1 (E). (* *P*<0.05, ns not significant). HMGB1: high mobility group box 1; LPS: lipopolysaccharide; GLY: glycine; ATP: adenosine triphosphate; NSA: necrosulfonamide; LDL: LDH: lactate dehydrogenase; LPS: lipopolysaccharide; ATP: adenosine triphosphate

**Figure 7:**
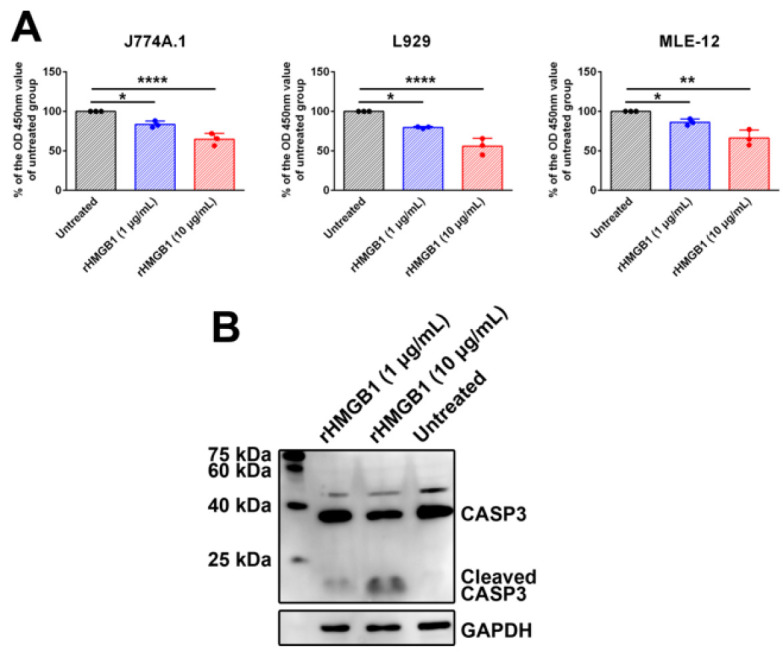
HMGB1 induced death of J774A.1 cells, MLE-12 cells, and L929 cells

(A) J774A.1 cells, MLE-12 cells, and L929 cells were treated with rHMGB1 (1 μg/ml or 10 μg/ml) for 8 hr. The viability of the cells was determined by CCK8 assays. rHMGB1 treatment resulted in a significant decrease in cell viability. (**** *P*<0.0001, ** *P*<0.01, * *P*<0.05) (B) Lysate of rHMGB1-treated J774A.1 cells was analyzed by western blot assay. rHMGB1 treatment resulted in the activation of CASP3. HMGB1: high mobility group box 1; rHMGB1: recombinant HMGB1

***Release of IL-1*** ***β*** ***and HMGB1 partially depends on GSDMD-mediated cell lysis***

Introducing glycine (to prevent pyroptotic cell lysis but not affect the formation of GSDMD pores) necrosulfonamide (NSA, an inhibitor that can abolish the formation of GSDME-NT oligomer by directly binding GSDMD) (18) into the experiment system remarkably reduced the concentration of IL-1β (Figure 6A) and HMGB1 (Figure 6B) in the culture medium of LPS+ATP-treated cells. Compared with NSA, GLY showed similar potency in inhibiting the production of HMGB1, but it was less potent in inhibiting the production of IL-1β (Figures 6A and 6B). Meanwhile, GLY treatment only resulted in a slight decrease in the mRNA level of IL-1β (Figure 6C), and NSA did not significantly affect the mRNA level of HMGB1 (Figure 6D). These results indicated that the inhibition of pyroptotic lysis of cells partially inhibited the release of IL*-*1β and almost completely abolished the release of HMGB1.

***HMGB1 induced cell death of myeloid and non-myeloid cells***

In this work, rHMGB1 (1 and 10 μg/ml) was used to treat J774A.1 cells, L929 cells, and MLE-12 cells. CCK8 assay revealed that 10 μg/ml rHMGB1 caused a significant decrease in cell viability (Figure 7A). The activation of caspase-3 in rHMGB1-treated J774A.1 cells was observed by Western blot assay (Figure 7B). The results indicated that HMGB1 could induce cell death of myeloid cells and non-myeloid cells, which might be attributed to apoptosis.

## Discussion

GEN is a potential candidate for anti-inflammatory drug development; many studies have shown the alleviation of inflammation-related diseases in animals receiving GEN treatment (19-21). Those studies confirmed the suppression of the expression of pro-inflammatory cytokines in animal models and/or in cultured cells by GEN. However, most of the studies reported that the suppression of pro-inflammatory cytokine expression by GEN is attributed to the inhibition of the transcription of pro-inflammatory cytokine mRNA (22, 23). Whether GEN disrupts the expression of pro-inflammatory cytokines by targeting the post-translation stage of cytokine expression is unclear. The current study determined that GEN could affect the release of IL-1β and HMGB1 by inhibiting CASP1/GSDMD-mediated pyroptotic cell lysis, which contributed to the inhibition of the expression of the two pro-inflammatory substances.

We first determined the inhibitory effect of GEN on the cleavage of GSDMD and the expression of pro-inflammatory substances in LPS-challenged mice. Intraperitoneal injection of LPS caused the cleavage of GSDMD in the liver and lung of mice. LPS challenge also caused a decrease in the amount of full-length GSDMD in the kidneys of mice. Administration of GEN completely abolished the cleavage of GSDMD caused by LPS. Mice that received GEN treatment had a lower serum concentration of pro-inflammatory substances such as IL-6, TNF-α, IL-1β, and HMGB1 than mice that did not receive GEN treatment. The inhibitory effect of GEN on the expression of pro-inflammatory substances and the cleavage of GSDMD was also investigated in J774A.1 cells. When the cells were treated with GEN before the LPS+ATP challenge, the increase in IL-6, TNF-α, IL-1β, and HMGB1 caused by the LPS+ATP challenge was suppressed at the mRNA and protein levels. When GEN was added after LPS priming, the mRNA level of the pro-inflammatory substances was not significantly affected, whereas the concentration of IL-1β and HMGB1 in the culture supernatant decreased significantly. These results indicated that GEN did not only affect mRNA transcription but also the post-transcriptional events in the production of these substances. Western blot assays showed that LPS+ATP treatment resulted in CASP1 activation and GSDMD cleavage in J774A.1 cells, but GEN inhibited these events in a dose-dependent manner. Given that GSDMD-mediated pore formation and pyroptotic cell lysis are crucial for the release of IL-1β and HMGB1 (24, 25), the current study speculated that GEN might inhibit the expression of pro-inflammatory substances by affecting the release of some pro-inflammatory substances. 

A recent study showed that estrogen receptor α (ERα), whose expression is up-regulated by GEN stimulation (26), can bind to GSDMD, thereby inhibiting GSDMD-mediated pyroptosis (27). The study also confirmed the inhibitory effect of GEN on NLRP3–GSDMD-mediated pyroptosis in mice (27). The current study demonstrated that the inhibition of GEN to the NLRP3–GSDMD pathway was attributed to the suppression of LPS+ATP-induced loss of intracellular K^+^ and overproduction of ROS. Our findings were consistent with the results of previous studies that showed GEN can inhibit K^+^ channels of cells (28-30), outward K^+^ current (30), low K^+^-dependent apoptosis of cells (31), and ROS production but promote ROS scavenging (32). Reduction of the intracellular K⁺ concentration (33) and burst of ROS (34) have been reported to activate the NLRP3 inflammasome via NEK7 (35). The NLRP3 inflammasome is a platform for CASP1 activation (36), and CASP1 is a protease that can efficiently activate the pyroptosis-mediating capacity of GSDMD (37). Therefore, the current study confirmed that GEN could exert its pyroptosis inhibitory effect, at least partially, by targeting the upstream events of inflammasome activation.

H&E staining showed that LPS induced the infiltration of inflammatory cells; cell death; or structural damage of the lung, liver, and kidney of mice. GEN alleviated the histopathological changes. To determine the relationship between the released pro-inflammatory substances and histopathological changes, we used rHMGB1 to treat different cells. Our results showed that rHMGB1 treatment resulted in the death of J774A.1 cells, MLE-12 cells, and L929 cells. Western blot showed the activation of CASP3 in rHMGB1-treated J774A.1 cells, which indicated the occurrence of apoptosis. Previous studies showed that excessive amounts of extracellular HMGB1 might cause tissue damage and organ dysfunction (38). Our results indicated that the released HMGB1 might lead to tissue damage by inducing cell apoptosis. Previous studies showed that excessive amounts of extracellular HMGB1 up-regulate the expression of pro-inflammatory substances in cells (39). This result might cause the formation of a positive feedback loop, leading to an increased production of pro-inflammatory substances and aggravated inflammation-related tissue damage. Therefore, the protective effect of GEN on LPS-induced tissue damage might be attributed to the suppression of HMGB1 release.

Daidzein is another isoflavone. A previous study showed that a synthesized daidzein derivative is more effective than daidzein in activating NLRP3 inflammasome and CASP1 and enhancing the activity of superoxide dismutase to scavenge ROS (40). Therefore, modification of GEN to obtain potent inhibitors of pyroptosis could be valuable for therapeutic efforts in some inflammation-related diseases.

## Conclusion

This study demonstrated that GEN could inhibit the release of pro-inflammatory substances by suppressing CASP1/GSDMD-mediated pyroptotic cell lysis, thereby alleviating the following harmful events caused by these substances. The inhibitory effect of GEN on the CASP1/GSDMD pathway was attributed to the inhibition of K^+^ loss and/or ROS production of cells. This study provides a basis for elucidating the mechanism by which GEN alleviates inflammation-related diseases.
